# The Bridge Between Classical and “Synthetic”/Chemical Psychoses: Towards a Clinical, Psychopathological, and Therapeutic Perspective

**DOI:** 10.3389/fpsyt.2019.00851

**Published:** 2019-11-20

**Authors:** Laura Orsolini, Stefania Chiappini, Duccio Papanti, Domenico De Berardis, John M. Corkery, Fabrizio Schifano

**Affiliations:** ^1^Psychopharmacology, Drug Misuse and Novel Psychoactive Substances Research Unit, School of Life and Medical Sciences, University of Hertfordshire, Hatfield, United Kingdom; ^2^Neomesia Mental Health, Villa Jolanda Hospital, Jesi, Italy; ^3^Polyedra, Teramo, Italy; ^4^NHS, Department of Mental Health, Psychiatric Service of Diagnosis and Treatment, Hospital "G. Mazzini", Teramo, Italy; ^5^Department of Neuroscience, Imaging and Clinical Science, Chair of Psychiatry, University of "G. D’Annunzio", Chieti, Italy

**Keywords:** psychosis, synthetic psychosis, chemical psychosis, novel psychoactive substances, NPS

## Abstract

The critical spread and dissemination of novel psychoactive substances (NPS), particularly among the most vulnerable youngsters, may pose a further concern about the psychotic trajectories related to the intake of new synthetic drugs. The psychopathological pattern of the “new psychoses” appears to be extremely different from the classical presentation. Therefore, clinicians need more data on these new synthetic psychoses and recommendations on how to manage them. The present mini-review aims at deepening both the clinical, psychopathological features of synthetic/chemical NPS-induced psychoses and their therapeutic strategies, according to the different NPS classes implicated, by underlining the main differences with the “classical” psychoses. A comprehensive review was conducted using the PubMed/Medline database by combining the search strategy of free-text terms and exploding a range of MESH headings relating to the topics of novel psychoactive substances and synthetic/chemical psychoses as follows: {(*Novel Psychoactive Substances*[Title/Abstract]) AND *Psychosis*[Title/Abstract])} and for each NPS categories as well, focusing on synthetic cannabinoids and cathinones, without time and/or language restrictions. Finally, an overview of the main clinical and psychopathological features between classical versus NPS-induced chemical/synthetic psychoses is provided for clinicians working with dual disorders and addiction psychiatry. Further insight is given here on therapeutic strategies and practical guidelines for managing patients affected with synthetic/chemical NPS-induced psychoses.

## Introduction

During the last decade, the “traditional” drug panorama has been gradually “reshaped” and integrated, even though not totally replaced, by the appearance of “new/novel psychoactive substances” (NPS) which are either newly created or existing substances which are now being used in “novel” modalities (1–3). The clinical, toxicological, and psychopathological effects of NPS have not been completely investigated and discovered through modelling; hence, clinical and psychopathological/psychiatric concerns have arisen among clinicians/professionals working in dual diagnosis, drug addiction, and mental health area (1, 2, 4–6). The recent published report from the European Monitoring Centre for Drugs and Drug Addiction (EMCDDA) described more than 730 NPS notified to the agency by the end of 2018, with 55 new compounds reported for the first time in Europe only in 2018 (3). Of these, 51% were synthetic cannabinoids (SC), 24% synthetic cathinones/“bath salts,” 5% benzodiazepines, 2% synthetic opioids, and 18% other substances (i.e., tryptamines, phenethylamines, arylcyclohexylamines, psychostimulants, etc.) (3).

SC/“Spice drugs,” mainly marketed as nail polish remover, deodorizers, incense, and potpourri, labeled as “natural”/“legal” alternatives to cannabis, have been documented to be dramatically associated with extremely more severe adverse health effects compared to "classical" cannabis (1, 7). SC are mainly consumed by inhalation, typically smoked together with a dried herbal material onto which they are previously sprayed during the production phase with chemical additives, or by e-cigarettes, or sold in tablets, etc. (1, 7). SC are mainly sold and purchased in “smart-shops” and on online drug marketplaces (7). Among these adverse effects typically described are: cases of fatalities, cardiac dysrhythmias, seizures, liver toxicity, kidney failure, hypothermia, hypertension, myocardial infarction, cardiac arrest, acute tubular necrosis, interstitial nephritis, rhabdomyolysis, nausea, vomiting, cognitive deficits, memory loss, sedation, catatonia, agitation, irritability, sympathomimetic syndrome, subarachnoid hemorrhage, cerebrovascular accidents, hematuria, bloody noses, bleeding gums, internal hemorrhage, hemoptysis, panic attacks, anxiety, altered mental status, coma, and a peculiar “synthetic” psychosis, designated "Spiceophrenia" (1, 7). Moreover, animal studies demonstrated that the administration of SC to adolescent rodents or non-human primates may determine the onset of a schizophrenia-like phenotype in adulthood (8–11).

Being beta-keto-phenethylamines, synthetic cathinones (i.e., ethylone and methylone) are structurally similar to amphetamines [i.e., 3,4-methyl-enedioxy-methamphetamine (MDMA), methamphetamine, etc.] and catecholamines, with subtle differences which modify their chemical, pharmacokinetic, and pharmacodynamic properties (1). Some cathinones are analogues of pyrovalerone, e.g.,3,4-methylenedioxypyrovalerone (MDPV), naphyrone, 3, 4-methylenedioxy-α-pyrrolidinobutiophenone (MDPBP), and α-pyrrolidinovalerophenone (α-PVP) (12). Different synthetic cathinones may exert different effects and potency levels on the dopaminergic, noradrenergic, and serotoninergic pathways, even though all usually own sympathomimetic and/or amphetamine-like effects (1). They are typically sold as pills, capsules, and powders, commonly insufflated (snorted/sniffed), orally consumed by “bombing” (swallowing the powder wrapped in a cigarette paper), mixed in a drink, or injected intravenously (13). Synthetic cathinones are generally known as “bath salts” in the USA and as “plant food” in Europe (1). Several motivations have been identified in their consumption and “appeal”/attractiveness among NPS and drug consumers, such as attaining feelings of euphoria/stimulation, increased energy, mood improvement, increasing empathy/openness, reaching a more mental clarity, experiencing vivid hallucinations, and increasing libido. Synthetic cathinone users seem to frequently report hyperthermia, rhabdomyolysis, renal failure, seizures, as well cardiac, psychiatric, and other neurological signs, in addition to the onset of several levels of agitation, ranging from mild agitation to severe psychosis (14). Moreover, mood instability and paranoid ideation have been described among chronic users (15–17). Furthermore, most synthetic cathinone consumers described tolerance, dependence, and withdrawal syndrome (1).

The class of synthetic hallucinogens/psychedelics includes all substances able to alter consciousness (i.e., synthetic lysergamides, tryptamines, and phenethylamines) by distorting time/motion/color/sound perceptions of consumers and/or altering the perception of “self” and/or stimulating some sensory/perceptual disturbances (1, 18, 19). Hallucinogens are usually consumed orally, occasionally through small blotter paper portions (i.e., “tabs”) held in the mouth to allow absorption through the oral mucosa. Moreover, it has been reported as route of administration by insufflation, smoking, rectal, and injection (intravenous and intramuscular) (1, 18, 19). The route of administration may influence the effects, their onset, and duration (1, 18, 19). Hypertension, tachycardia, hyperthermia, dizziness, sleeplessness, loss of appetite, xerostomia, sweating, impulsiveness, fast and labile emotional modifications (from fear, anxiety to euphoria), numbness, weakness, and tremors have been reported as frequent short-term effects. While long-term effects may comprise persistent psychosis (i.e., visual disturbances, disorganized thinking, and paranoia), mood fluctuations, and/or onset of a hallucinogen-persisting perception disorder (HPPD) (1, 5, 19).

Synthetic/novel psychostimulants comprise several NPS classes, i.e., piperazines (compounds labeled as “legal” alternatives to ecstasy, mainly identified as cutting agents in some ecstasy pills) and “amphetamine-type stimulants” (ATS) (1, 3). ATS, e.g., PMA (4-methoxyamphetamine/“Dr. Death”), PMMA (4-methoxymeth-amphetamine), 4-MTA (4-methylthioamphetamine/“flatliners”), DMA (2,5-dimethoxyamphetamine), aminorex derivatives (4,4″-DMAR); diclofensine, methiopropamine, etc., have been marketed as para-substituted methoxy drugs pharmacologically and clinically comparable to amphetamines (1, 3, 18). ATS are commonly supplied as tablets/crystals/powder, sometimes mixed with other substances (20). They are usually swallowed/smoked/snorted, and their common “street names” include “crystal meth” (aka “ice”)/“speed"/“crystal,” etc. (20). Obsessive delusions, paranoia, hallucinations, suicidal ideation, anxiety, insomnia, depression, and psychotic symptoms have been commonly reported following intake of ATS (20, 21).

Overall, NPS have been largely associated with several clinical and psychiatric symptomatology which may vary from the occurrence of an acute transient psychotic episode to more complex psychopathological patterns, depending on the substance implicated (and its pharmacological profile), the frequency, intensity and route of administration, and vulnerability and individual characteristics of NPS consumers (1, 4–6, 22, 24) (as shown in **Table 1**). The psychopathological pattern of NPS-induced “new/synthetic psychoses” appears to be extremely different from the classical presentation, by introducing a new interesting field of research from a psychopathological and phenomenological point of view. More specifically, NPS mainly implicated in the onset of psychotic patterns are represented by SC, synthetic cathinones, psychostimulants, and some psychedelics/hallucinogenic NPS (1, 6, 19). However, as synthetic cannabinoids and cathinones are the most representative NPS groups, the present review mainly focused on these two categories in collecting, analyzing, and critically discussing data so far published about NPS-induced synthetic/chemical psychoses by underlining differences with “classical” psychosis.

**Table 1:** Pharmacological and clinical features of the main NPS classes.

Type of NPS category	Description	Neurotransmitter/pathways/receptors implicated	Clinical manifestations
Psychostimulants	This category includes all compounds derived from psychostimulants (i.e., amphetamine, methamphetamine, MDMA, and cocaine). Some synthetic psychostimulants include the biochemical family of synthetic cathinones (see below) and novel stimulants, aminorex derivatives, ATS such as diclofensine, methiopropamine, 4,4′-DMAR, NBOMe-series, 2C-series, etc.	Traditionally, psychostimulants primarily target monoaminergic systems, leading to increased extracellular levels of SER, DA, and/or NA.	*Desired effects*: Euphoria Social disinhibition Extroversion Increased energy Appetite suppressant “High”
			*Untoward effects*: Serotoninergic syndrome Psychosis Paranoid ideation Impulsivity Mania Agitation Cardiovascular symptoms Hyperthermia
Synthetic cathinones ("bath salts")	This category includes analogues of naturally occurring cathinones such as *Catha edulis* (Khat). Synthetic cathinones are classified into: a) Substrates of DAT, SERT, and NAT (pharmacological profile similar to MDMA: i.e., butylone, ethylone, 4-MEC, etc.) b) Selective substrates of DAT (pharmacological profile similar to amphetamine and methamphetamine, i.e., methcathinone, flephedrone, etc.) c) Non-substrates of transporter inhibitors (i.e., MDPV, etc.) Some synthetic cathinones include mephedrone ("meow meow"), methedrone, methylone, 3-MMC, 2-MeOMC, 4-MeO-a-PVP, 4-MeO-PBP, 4-MeO-PV9, 4-MPD, 4F-PV8, 4F-PV9, 4F-PVP, a-PBT, a-PHP, a-PVT, dibutylone, DL-4662, Ethylone, MDPPP, MOPPP, NEB, Pentedrone, PV-8 (crystals), etc.	The mechanism by which synthetic cathinones exhibit an effect is similar to other stimulants through functionally changing monoamine transporters. They inhibit monoamine transporters, leading to increased extracellular levels of SER, DA, and NA. They exhibit a sympathomimetic activity.	*Desired effects*: Euphoria Hypervigilance Increased energy Openness Empathy Increased libido Appetite suppression
			*Untoward effects*: Excited delirium Hallucinations Agitation Aggressiveness Paranoid ideation Exacerbation of mood disorders
Synthetic cannabinoids (synthetic cannabimimetics, synthetic cannabinoid receptor agonists, "Spice drugs," "legal cannabis alternative")	A wide range of brands of herbal products containing synthetic cannabinoids have been available on the market. They include, but are not limited to, Spice, Black Mamba, Annihilation, and Amsterdam Gold. New brands continue to emerge. Brand names are sometimes reminiscent of street names of strains of cannabis. Synthetic cannabinoids may be classified into seven major structural groups: naphthoyndoles (e.g. JWH-018, JWH-073, etc.); naphthylmethylindoles; naphthoylpyrroles; naphthylmethylindenes phenylacetylindoles (i.e. benzoylindoles, e.g. JWH-250); cyclohexylphenols (e.g. CP 47,497 homologues of CP 47,497)\ classical cannabinoids (e.g. HU-210).	They act on the endocannabinoid system, particularly on: CB_1_ (total agonism) CB_2_ (total agonism)	*Desired effects*: Relaxation Analgesia “High” Sedation Euphoria Anxiolysis
			*Untoward effects*: Paranoid ideation Brief psychotic episode Persistent psychotic disorder/“Spiceophrenia” Cognitive impairment Anxiety/agitation Dyscontrol of impulses Dysphoria Manic symptoms Worsening of preexisting bipolar disorder and/or psychosis and/or schizophrenia Seizures Tachycardia, hypertension, mydriasis, hyperglycemia, hypokalemia, dyspnea, tachypnea, nausea, vomiting Stroke, encephalopathy, renal failure Serotoninergic syndrome
Dissociatives	The compounds included in this category produce dissociative effects similar to phencyclidine (PCP or “angel dust”), ketamine (“Special K” or “K” or “Ket”), methoxetamine etc. Some of NPS included in this category comprise 3-MeO-PCP, ethylketamine, 3-HO-PCP, 4-MeO-PCP, Diphenidine, Methoxphenidine, etc.	Dissociative drugs act mainly as uncompetitive antagonists, through open channel blockade, of the glutamate ionotropic NMDA receptor. Dissociatives affect numerous other receptors (i.e., DA, opioid, 5-HT, adrenergic, nicotinic, muscarinic, and adenosine receptors) and ionic channels, leading to their occasional informal description of being neurochemically “dirty drugs.”	*Desired effects*: Dissociation/depersonalization (at high dosages) Analgesia “High” (at low dosages) Euphoria (at low dosages) “Out-of-body” or “near-death” experiences Weightlessness
			*Untoward effects*: Headache Psychosis Reference ideation Hallucinations Flashbacks Nausea Dizziness Paranoia Anxiety Cognitive impairment Serotonergic syndrome (possible)
Hallucinogens (psychedelics/psychotomimetics/entheogens)	A category of substances developed as analogues of “classical” hallucinogens (i.e., LSD, psilocybin). They can be further divided into three subgroups: Phenethylamines (e.g., mescaline, Bromo-DragonFLY, etc.) Tryptamines (e.g., psilocybin, 5-MeO-DALT, etc.) Lysergamines (e.g., LSD, AL-LAD, etc.) Novel tryptamines recently marketed include 5-MeO-DMT, 5-MeO-DIPT, 4-HO-DALT, 5-MeO-AMT, DET, etc. Among the novel derivatives of “classical” psychedelic phenethylamines/MDMA-like drugs, we include the following: 2C-molecules (e.g., 2-CB/Nexus, 2C-I, 2C-E, etc.), PMA/Dr Death, Bromodragonfly/B-fly, 25C-NBOMe/N-bomb/Pandora, etc. Some hallucinogens also include herbs/plants such as *Salvia divinorum*, Mescaline, or other herb-related compounds previously used in traditional shamanic practices.	All three subgroups share a common mechanism on serotonergic system, represented by the agonism/partial agonism at the 5-HT_2A_ receptor (activation), 5-HT_1A_, 5-HT_2C_. However, the different hallucinogenic/psychedelic drugs may differently interact with other neurotransmitters, i.e., NMDA receptors, σ-receptors, µ-opioid receptors, and muscarinic receptors, apart from causing serotonin and dopamine reuptake inhibition at their transporters.	*Desired effects*: Perceptual restructuralization Oceanic boundlessness Visual hallucinations Alterations in sensory perception Distortion in body image Depersonalization Entactogenic feelings Euphoria
			*Untoward effects:* Anxiety Agitation Psychosis Hallucinations Serotonergic syndrome (possible)
Sedatives/analgesics	This category includes the synthetic/derivatives opioids (i.e., AH-7921, IC-26, MT-45, nortilidine, W15, W18, U-47700, lefetamine, etc.) and designer/synthetic benzodiazepines (i.e., etizolam, flubromazepam, flubromazolam, phenazepam, pyrazolam).	They may act on opioid system and/or GABA system.	*Desired effects*: Analgesia Sedation Emotional
			*Untoward effects*: Lack of consciousness Somnolence/sedation Respiratory depression Coma and death

ATS, amphetamine-type stimulants; SER, serotonin; DA, dopamine; NA, noradrenalin; SERT, serotoninergic transporter; DAT, dopamine transporter; NAT, noradrenergic transporter; NMDA, N-methyl-D-aspartate.

## Material and Methods

### Search Sources and Strategies

A mini-review was conducted by analyzing and collecting clinical data (only human studies) on cases of NPS-induced psychosis, by using PubMed/Medline and focusing on synthetic cannabinoids and cathinones. We combined the search strategy of free-text terms and exploded MESH headings for the topics of “Novel Psychoactive Substances” and “Synthetic/Chemical Psychoses” as follows: {(*Novel Psychoactive Substances*[Title/Abstract]) AND *Psychosis*[Title/Abstract])} and for each NPS category (synthetic cannabinoids and synthetic cathinones) as well, without time/language restrictions. All studies published up to 30 June 2019 were included. In addition, secondary searches were performed using the reference listing of all eligible as well as relevant articles and consultation with experts in the field and or manual searches.

### Study Selection, Data Extraction, and Management

We considered studies evaluating the relationships between NPS and synthetic psychosis. We examined all titles and abstracts and obtained full texts of potentially relevant papers. Working independently and in duplicate, two reviewers (LO and SC) read the papers and determined whether they met inclusion criteria. Duplicate publications were excluded. All experimental and observational study designs, including case reports, case series and surveys, were retrieved. Narrative and systematic reviews, letters to the editor, and book chapters were excluded, even though they were used for retrieving further secondary searches. To be included in the present review, studies were required to meet the following criteria: a) empirical and peer-reviewed study; b) at least an abstract with estimates and/or full results published in English; c) human studies; and d) provide data on synthetic psychosis induced by NPS. LO and SC independently extracted the data on participant characteristics, intervention details, and outcome measures. Disagreements were resolved by discussion and consensus with a third member of the team (GDP). Data were collected using an *ad hoc* developed data extraction spreadsheet.

### Characteristics of Included Studies

The set of keywords initially generated 402 results. A total of 123 papers were excluded because of duplication; seven papers were excluded through lack of an english abstract. Of the remaining 272 studies, 145 studies were excluded because they did not meet the inclusion criteria or because they were animal studies. Of the remaining 127 papers, 37 papers were excluded because they were reviews, letters to editors, or meta-analyses, while seven papers were not included here due to the lack of an available full text or an abstract useful for collecting relevant data. Finally, a total of 83 papers were included and accounted for in our analysis. **Table 2** clearly explains the main characteristics (study design, sample size, main outcomes, and findings) of all studies retrieved.

**Table 2:** Summary of included studies.

Reference	Study design (country)	Sample features (size, age, ethnicity, sex)	Psychiatric history	NPS involved	Symptomatology	Previous substance use history	Treatment [drug name, dosage, duration (if available)]
Studies on NPS-induced psychosis in general (unspecified or multiple NPS involved)							
Stanley et al. (25)	Cross-sectional survey (UK)	86/388, 36.1 ± 9.4; NA, 60 M and 26 F	Personal psychiatric history of schizophrenia, schizoaffective disorder, and personality disorders	NPS unspecified	Psychosis	NPS users present significantly higher levels of cannabis, alcohol, and substitute opiate use	General adult psychiatric ward (NA, NA, NA)
Vallersnes et al. (26)	Retrospective cohort study—European Drug Emergencies Network (Euro-DEN, several countries)	5,529, 24-38, NA, 79.3% M	NA	MDPV (27.3%), tryptamines (57.1%), methylphenidate (23.1%), SC (15.4%), mephedrone (5.7%), methedrone (3.3%)	Psychosis, significantly associated with agitation (63.2%; *p* < .001); hallucinations (43.7%, *p* < .001) and anxiety (37.1%, *p* < .001)	NA	ED (NA, NA, NA)
Liakoni et al. (27)	Retrospective cohort study (Switzerland)	157,328, 16–74, NA, 68% M	NA	2 cases with NPS (2C-P and methylone)	Psychosis	NA	ED (NA, NA, NA)
**Studies on synthetic cannabinoid-induced psychosis**							
Every-Palmer (28)	Case report (New Zealand)	NA, NA, NA, NA	Personal psychiatric history of psychotic disorder (*n* = 5)	“Aroma” blend, containing CP-47,497 and/or JWH-018 (SC)	Mental states deteriorated significantly after use including sudden re-emergence of florid psychosis, predominantly agitation, disorganization, and delusional beliefs (*n* = 5)	NA	NA
Müller et al. (29)	Case report (Germany)	1, 25, NA, M	Personal psychiatric history of cannabis-induced recurrent psychotic episodes associated with a strong family history of paranoid schizophrenia	Spice (SC)	Anxiety, psychotic symptoms with feelings of manipulation, worsening of symptomatology (imperative voices and paranoid hallucinations)	Cannabis	NA
Rodgman and Kinzie (30)	Case report (USA)	3, NA, NA, NA	NA	"Mojo" (SC)	Acute psychosis	NA	Beta-blockers (NA), low dose of BDZs (NA)
Sobolevsky et al. (31)	Case report (Russia)	3, 22 ± 1, NA, 2 M and 1 F	NA	Tropical Synergy (SC) containing JWH-018, CP 49,497	Anxiety, tachycardia, paranoia, hallucinations, short-term memory, and sense of time impaired	NA	NA
Vearrier and Osterhoudt (32)	Case report (USA)	1, 17, NA, F	None	JWH-018 (SC)	Violent and “crazy” visual hallucinations, lower extremities numbness, muscle twitches, elevated pulse, dilated pupils, anxiety	Occasionally abuse alcohol and cannabis	ED, LOR (2 mg ev, once)
Benford and Caplan (33)	Case report (USA)	1, 20, NA, M	None	Spice (SC)	Severe anxiety and paranoia, auditory and visual hallucinations, halted speech	Cannabis	ED
Castellanos et al. (34)	Case series (USA)	11, 15–19, NA, 10 M and 1 F	Personal psychiatric history of ADHD (*n* = 3); ADHD + learning disorder + MDD (*n* = 1); none (*n* = 7)	SC	Euphoria, irritability, anxiety, numbness, memory impairment, auditory and visual hallucinations, paranoia, palpitations, muscle tremors, weakness, blackouts, restlessness, stimulation	Cannabis and alcohol use (*n* = 10)	NA
Every-Palmer (35)	Case series (New Zealand)	9/15, 18–65, NA, M	Personal psychiatric history of schizophrenia (*n* = 10), schizoaffective disorder (*n* = 4), bipolar disorder with psychotic features (*n* = 1)	JWH-018 (SC)	Excited psychotic symptoms, relapse of psychosis (agitation, delusions and disorganization after SC intake), anxiety, paranoia	Cannabis	NA
Forrester et al. (36)	Retrospective cohort study (USA)—Texas Poison Center Network (2010)	464, 12–67, NA, 343 M and 118 F	NA	SC	Agitation (18.5%), hallucinations (10.8%), drowsiness (18.5%), vomiting, nausea, tachycardia	Alcohol, cocaine, acetaminophen, hydrocodone, chlorpeniramin, dextromethorphan, carisoprodol	Hospitalization, ev fluids, BDZs, oxygen, antiemetics, AC (NA)
Hurst et al. (37)	Case series (USA)	10, 21–25, NA, M	None	SC	Auditory hallucinations, paranoid delusions, blocked thoughts, disorganized speech and behavior, suicidal ideation, alogia, ideation, insomnia, flatted affect, psychomotor retardation and/or agitation, anxiety	Concurrent cannabis or alcohol use (*n* = 8)	Hospitalizations (*n* = 7), not specified AP (*n* = 10) (NA)
Johnson et al. (2011)	Case report (USA)	1, 23, Caucasian, M	None	Spice (SC) smoked 96 and 48 h before admission	Paranoia, disorganized thoughts, nonsensical speech, delusion	Earlier cannabis use over 3 years ago	Spontaneous remission after 24 h
Locatelli et al. (39)	Retrospective cohort study (Italy)—Pavia Poison Centre and National Early Warning System for Drugs	17, 14–55, Caucasian, NA	NA	“Spice” (*n* = 1), “N-Joy” (*n* = 6), “Forest Green” (*n* = 3), “Jungle Mystic Incense” (*n* = 5), JWH (*n* = 2), all SC	Agitation, confusion, hallucinations, dyspnea, coma, seizure, mydriasis, xerostomia, vertical nystagmus, psychomotor agitation, vomiting	NA	BDZs (NA), symptomatic treatment
McCain et al. (40)	Retrospective cohort study (USA)—Arkansas Poison and Drug Information Center (March–August 2010)	6/9, NA, NA, NA	NA	SC	Agitation/irritability (50%), hallucinations (33.3%), confusion (66.7%), nausea (33.3%), mydriasis (33.3%), pallor (33.3%), tachycardia (100%), hypokalemia (100%)	NA	BDZs (NA), supportive infusive therapy
Simmons et al. (41)	Case report (USA)	a) 1, 25, NA, M b) 1, 21, NA, M c) 1, 19, NA, M	None	"Spice” (SC) containing JWH-018, JWH-073	a) Seizure, tachycardia, acidosis, unresponsiveness to verbal stimuli, "eye-crossed and flailing arms", mydriasis b) Hypertension, unresponsiveness, agitation, c) Delusions, paranoia, short-term memory impairment, bizarre behavior, muscle spasm, depressed breathing	None	BDZs (LOR, 4 mg, NA), APs (HALO, 5 mg, NA), supportive infusive therapy and observation
Young et al. (42)	Case report (USA)	1, 17, NA, M	None	“K9” smoking (SC)	Hallucinations, dizziness, tachycardia, chest pressure that lasted for 3 days, difficulty breathing, lightheadedness	Occasional alcohol use	NA
McGuiness and Newell (43)	Case report (USA)	1, 18, NA, F	NA	SC	Paranoia, chest pain, hyperventilation, nausea, panic attack, ideas of reference	NA	NA
Tung et al. (44)	Case report (Hong Kong)	1, 36, Chinese, M	Family psychiatric history of SMI (unspecified) and SUD	“K2” (SC) daily for 4 weeks before admission	Agitation, profuse seating, tachycardia, delusion, elevated blood pressure, irritability, insomnia, restlessness, accelerated speech	Previous abuse history of multiple psychoactive substances (codeine, heroin, and other unspecified)	Hospitalization; spontaneous remission
Van der Veer and Friday (45)	Case report (USA)	3, 20–30, NA, M	Personal psychiatric history: none (*n* = 1), PTSD (*n* = 1), amphetamine-induced brief psychotic episodes (*n* = 1)	“Spice” (SC) 3–4 weeks before admission	Persistent psychosis after SC intake, disorganized speech, poverty of thought, loosening of associations, paranoia, delusion, aggressiveness, inappropriate affect, suicidality, "Capgras delusions"	Previous cannabis use	Hospitalization; HALO (*n* = 2, NA, NA), RIS (*n* = 1, NA, NA)
Bebarta et al. (46)	Case report (USA)	3, 19–23, NA, 2 M and 1 F	NA	“Spice” (*n* = 1) and “Space” (*n* = 2), all containing SC	Paranoia, hallucinations, agitation, tachycardia, hyperglycemia, mild drowsiness, short-term memory impairment, hyperglycemia, anxiety, agitation, breathing difficulty, hyperventilation, tachycardia, injected sclera	None	ED observation; LOR (2 mg, NA)
Cohen et al. (47)	Case report (USA)	3, 16–18, NA, 2 M and 1 F	NA	SC	Catatonia, tachycardia, vertical nystagmus, agitation, aggression restlessness, tachycardia, hyperventilation, disorientation, slowed speech	NA	NA
Hoyte et al. (48)	Retrospective Cohort study (USA)—National Poison Data System (January–October 2010)	1898, 20 (median), NA, 74.3% M	NA	“K2” and “Spice Gold”, all containing SC	Hallucinations, delusions (9.4%)	NA	BDZs (*n* = 16%, NA, NA)
Oluwabusi et al. (49)	Case report (USA)	2, 16–17, Hispanic, M	None	“K2” and “Spice”, all containing SC	New-onset psychosis, insomnia, hyperactivity, anxiety, paranoid and grandiose delusions, musical auditory hallucinations, colorful visual hallucinations, low mood, agitation, irritability, pressure of speech, flight of ideas, disorganized behavior, thought broadcasting, somatic preoccupation, mild cognitive impairment	NA	Hospitalization, ARI (*n* = 1, 20 mg daily, NA), OLA (*n* = 1, 15 mg daily, NA)
Peglow et al. (50)	Case report (USA)	1, 59, Latin, M	PTSD	Smoking two joints of “Spice” (SC) daily for the previous 3 weeks before admission	New-onset psychosis, visual hallucinations, disorganized and bizarre behavior, consciousness disorder, flashbacks of trauma	Alcohol, heroin, cocaine, cannabis discontinued 3 years before	Hospitalization, BZDs (NA, NA, NA), gabapentin (400 mg QID), hydroxyzine (25 mg TID), ARI (10 mg qD), benztropine (1 mg BID), BUP (150 mg BID)
Vandrey et al. (51)	Internet-based survey (13 different countries)	391, 26 ± 9, 90% Caucasian, 83% M	NA	“Spice” (SC)	Hallucinations (28%)	Alcohol (92%) Cannabis (84%)	NA
Barratt et al. (52)	Online questionnaire using purposive sampling strategy (Australia)	316, 23–34, NA, 77% M	NA	SC	Paranoia (18%) Psychosis (4%)	Cannabis (96%)	NA
Berry-Cabán etal. (53)	Case report (USA)	1, 20, Hispanic, M	None	“Spice” (SC)	New-onset psychosis, uncommunicative, unable to follow commands, combative	Alcohol, "plant food"	Hospitalization, LOR (2 mg, three administrations during hospitalization), HALO (5 mg once), RIS (1 mg daily, NA)
Chan et al. (54)	Case-report (UK)	1, 21, NA, M	None	6-APB (SC), metabolites of both THC and JWH-122 (SC)	Delusions (“trying to read his mind”, “looking at him in a funny way”), agitation, suicidal and homicidal tendencies, paranoia, low mood	Cannabis	Hospitalization, diazepam (4–10 mg, NA)
Hermanns-Clausen et al. (55)	Retrospective cohort study (Germany)—Database of the Poisons Information Center Frelburg (2008–2011)	29, 14–30, NA, 25 M and 4 F	NA	SC	Mild visual or auditory hallucinations, tachycardia, drowsiness, paresthesia, abdominal pain, mild hypoglycemia, mild electrolyte imbalance, short-term hypothermia, delirium, agitation, confusion, rhabdomyolysis, etc.	NA	Supportive care, potassium supplementation, BDZs (NA)
Papanti et al. (7)	Case report (Italy)	1, 18, Caucasian, M	None	“Bonzai” herbal blend (SC)	Anxiety, insomnia, palpitations, ideas of reference, somatic and visual hallucinations, autoscopy, agitation, behavioral dyscontrol	None	Hospitalization with diazepam 5 mg ev, then, bromazepam 3 mg daily and OLA 5 mg daily for 4 weeks
Glue et al. (56)	Case series (New Zealand)	17, 26.1 ± 10, NA, 10 M and 7 F	Personal psychiatric history of recurrences of preexisting affective disorders (*n* = 9/17)	“K2” (SC)	Paranoia, thought disorder, disorganized behavior, anxiety, depression, suicidal ideation	NA	Hospitalization, ADs (NA, NA), APs (NA, NA)
Leibu et al. (57)	Case report (USA)	1, 36, African-American, M	Personal psychiatric history of schizophrenia and cannabis dependence	“K2” (SC) 3 times smoked weeks before admission	Worsening paranoia, illogical speech and auditory hallucinations	Previous cannabis use	Hospitalization, ECT, CLO (225 mg BID, NA)
Bozkurt et al. (58)	Survey on sociodemographic and clinical data (Turkey)	158, 26.1 ± 7.1, NA, 94.9% M	Personal psychiatric history of comorbid psychiatric disorders (7%)	“Bonzai” (SC) in 70.3% “Jamaika” (SC) in 21.5%	Hallucinations (40.4%), delusions (16%)	Cannabis	NA
Celofiga et al. (59)	Case report (Slovenia)	4, 21–35, NA, M	Personal psychiatric history of schizophrenia	AM-2201 (SC)	Delusions, hallucinations (*n* = 2), psychosis relapse	None	None (*n* = 1), increased BDZs (*n* = 3)
Haro et al. (60)	Case report (Spain)	1, 18, NA, F	NA	SC for at least 6 months	New-onset psychosis, violent behavior, talking to self, visual hallucinations	Cannabis	ARI (15 mg daily, 2 months), LOR (NA, NA), Biperiden (NA, NA)
Meijer et al. (61)	Case report (USA)	1, 26, NA, M	Personal psychiatric history of ADHD stable with lisdexamfetamine	“Black Diamond” (SC)	New-onset psychosis, paranoid delusions	NA	Amputation
Smith and Roberts (62)	Case report (USA)	1, 17, NA, M	None	“K2” daily for 2 months before admission	Auditory and visual hallucinations, catatonia, disorganized thought process, new-onset psychosis	NA	Hospitalization, LOR (2 mg daily, NA), OLA (NA, NA), ECT (6 sessions, NA)
Durand et al. (63)	Case report (USA)	1, 23, NA, M	Family psychiatric history of schizophrenia	“Mr. Nice Guy” (SC) sporadically for 6 months	New-onset psychosis, persecutory delusions	Cannabis	Hospitalization; HALO (10 mg TID, NA), VA (500–1,000 mg, NA), LOR (2 mg TID, NA)
Schwartz et al. (64)	Case series (USA)	7, 16–30, NA, 3 M and 4 F	None	“Crazy Clown” (SC)	Delirium, new-onset psychosis, aggressive behaviors	1 subject with cocaine use	ICU, intubation (*n* = 3), no treatment (*n* = 3), normal saline (*n* = 1)
Ustundag et al. (65)	Case report (Turkey)	1, 18, NA, M	None	SC for 6 months	Talking to self and laughing, new-onset psychosis, delusions, manic symptoms	Volatile substances	OLA (10–20 mg daily, NA), VA (500–1,500 mg daily, NA), QUE (200–400 mg daily, NA)
Sönmez and Köşger (66)	Case report (Turkey)	1, 31, NA, M	None	“Bonsai” (SC) 3 times a week for >6 months	Anger, insomnia, delusions, new-onset psychosis	None	Hospitalization; OLA (NA)
Rahmani et al. (67)	Case report (USA)	a) 1, 17, Caucasian, M b) 1, 17, Caucasian, M	a) Strong family psychiatric history b) Strong family psychiatry history	“Spice” (SC)	a) Delusions, visual hallucinations, new-onset psychosis b) Paranoia, disorganized thought and behavior, new-onset psychosis	a) Cannabis, LSD, psilocybin, mushrooms, bath salts, oxycodone b) Cannabis, LSD, ecstasy, benzodiazepines	a) Hospitalization, CLO (NA), metoprolol (NA) b) Hospitalization, CLO (NA)
Tyndall et al. (68)	Retrospective cohort study (USA)	35, 14–58, NA, 88.6% M	NA	SC	Hallucinations (6%)	NA	Hospitalization (28%)
Altintas et al. (69)	Cross-sectional study (Turkey)	50/81, 25.9 ± 5.5, NA, M	Strong family psychiatric history and personal psychiatric history of substance use disorder (36%)	SC	Paranoia, disorganized behavior, suicide thoughts, anxiety, visual and auditory hallucinations	Cannabis	NA
Khan et al. (70)	Case report (USA)	a) 1, 21, African-American, M b) 1, 17, Caucasian, M	a) Personal psychiatric history of untreated childhood ADHD b) None	a) Continuous heavy use of "Kush" for the previous 18 months b) 2-week "Spice" binge	Catatonia, incongruent affect and paraphasic errors, flat affect, blank stare, echolalia, muscle rigidity, bradykinesias, psychosis, euphoria, grandiosity, paranoia, decreased sleep, disorganization	a) Occasional previous cannabis use b) Periodic cannabis smoking for 1 year prior	a) Hospitalization; ARI (25 mg daily, NA) b) Hospitalization; VA (100 mg qHS, NA), LOR (2 mg BID, NA), OLA (NA, NA)
Hermanns-Clausen et al. (71)	Retrospective cohort study (Germany)—Poisons Information Center (2011–2014) and Institute of Forensic Medicine of the University Medical Center Freiburg	22/46, 12–25, NA, 18 M and 4 F	NA	SC	Visual hallucinations (23%), perceptual disturbance (9.1%), retrograde amnesia (4.5%), tachycardia (82%), nausea (78%), somnolence (55%), mydriasis (45%), restlessness (13.6%), agitation (13.6%),	Alcohol (*n* = 1)	BDZs (*n* = 2, NA), metoprolol (*n* = 3, NA), metoclopramide (*n* = 2, NA), ondansetron (*n* = 2, NA), diphenhydramine (*n* = 2, NA), lamotrigine (*n* = 1, NA)
Bassir Nia et al. (72)	Retrospective cohort study (USA)	82/676, NA, NA, NA	NA	SC ± cannabis	Subject with SC use only were significantly associated with more psychotic symptoms (OR = 4.44), more psychotic disorder diagnosis (OR = 6.74), and greater agitation (OR = 4.39) and aggression (OR = 3.10), followed by subjects with SC and concomitant cannabis use (OR = 3.61, OR = 4.78, and OR = 2.51, respectively)	NA	APs, MS, AD (NA, NA)
Roberto et al. (73)	Case report (USA)	1, 18, African American, M	None	"K2" and "Spice" daily for 3–4 weeks	Insomnia, paranoid ideation, agitation, elated mood, beliefs that others were inserting thoughts into his head ("thought insertion" and "thought broadcasting"), pacing, bizarre delusional thoughts with thought derailment, disorganized behavior, decreased short-term memory	Alcohol and cannabis	Hospitalization; RIS (5 mg daily, NA), LOR (2 mg, NA)
Samaan et al. (74)	Case report (USA)	1, 18, Hispanic, M	None	SC	Acute-onset auditory hallucinations, paranoid delusions, panic attacks, palpation, shortness of breath, diaphoresis, chest tightness, hand numbness, impulsivity, aggressiveness, agitation, suicidal thoughts	Cannabis	NA
Ozer et al. (75)	Case report (Turkey)	1, 17, NA, M	None	Inhaled "Bonsai" (SC) for 10 days before admission	Capgras syndrome, persecutory delusions and auditory hallucinations	NA	Hospitalization; OLA (10 mg daily, 3 months)
Shalit et al. (76)	Retrospective cohort study (Israel)	60, 30.46 ± 7.83, NA, 86% M	NA	SC	Higher PANSS score in SC users vs. cannabis users No differences in major psychiatric diagnoses between SC vs. cannabis users	Cannabis (73.3%)	Hospitalization
Waugh et al. (77)	Retrospective cohort study (UK)—National Poisons Information Service telephone enquiry records (2007–2014)	510, 12–78, NA, 80.8% M	NA	"Black Mamba" (SC) in 20.3% "Pandora's Box" (SC) in 15% "Clockwork Orange" (SC) in 6.2%	Hallucinations (4.6%), paranoia (1.2%)	9% previous use of other substances	NA
Zorlu et al. (78)	Case–control (Turkey)	22 SC users vs. 18 HC, 26.5 ± 5.2, NA, NA	NA	SC	Long-term use of SC associated with white matter abnormalities and disturbed brain connectivity associated with an increased vulnerability to psychosis	NA	NA
Babi et al. (79)	Case report (USA)	1, 40, NA, M	None	SC	Acute psychosis, new-onset refractory status epilepticus	None	LOR ev (NA, NA), VA (NA, NA), levetiracetam (NA, NA), lacosamide (NA, NA)
Manseau et al. (80)	Retrospective cohort study (USA)	110, 33 (median), non-white (90%), 95.5% M	Personal psychiatric history of primary psychotic disorder diagnosis (40%)	SC	Acute psychotic symptoms (70%), agitation	Alcohol (39.1%), cannabis (35.5%), cocaine (24.5%)	Inpatient admission (34.5%), unspecified APs
Monte et al. (81)	Retrospective cohort study (USA)—Toxic Registry (2010–2015)	353, 25 (median), NA, 84% M	NA	SC	Delirium and toxic psychosis (41.4%), hallucinations (7.1%)	3.4% previous cannabis use whilst 5.7% previous use of other substances	BDZs (37%) and unspecified APs (10%) most frequently prescribed
Kassai et al. (82)	Qualitative research (Hungary)	6, 20–27, NA, M	NA	SC	Paranoia, difficulty socializing, increased egoism, self-neglect, switch off brain, inability to sleep, feeling under control, sweating	NA	NA
Van Hout and Hearne (83)	Online survey (UK)	6, NA, NA, 3 M and 3 F	NA	SC	Agitation, restlessness, fear, paranoia, aggression, severe dissociation, chest pain, aches and pains, palpitations, nausea, sweating, vomiting	NA	NA
Welter et al. (84)	Prospective pilot study (Germany)	24/332, 23.52 ± 5.29, NA, 71.4% M	Personal psychiatric history of schizophrenia, schizoaffective disorder and schizophrenic disorder (71.4%)	SC ± cannabis	SC use was 2 times higher among psychotic patients vs. non-psychotic patients (*p* = .033). Delusion of persecution, hallucinations, blunted affect, motor retardation, disorganization or worsening of preexistent psychotic symptoms	Cannabis	NA
Bonaccorso et al. (85)	Case series (UK)	a) 1, 28, Caucasian, M b) 1, 32, Caucasian, F c) 1, 20, Black-Caribbean, M d) 1, 39, Asian British, M	a) Personal psychiatric history of paranoid schizophrenia b) Personal psychiatric history of schizoaffective disorder, poly-substance misuse (cocaine and heroin) c) Personal psychiatric history of first psychotic episode post poly-substance misuse d) Personal psychiatric history of bipolar disorder	SC	Intense exacerbations of positive symptoms, psychomotor agitation, sexual disinhibition, verbal/physical aggression, poor responses to medications	Previous poly-substance misuse (variable)	a) RIS-LAI (37.5 mg fortnightly, NA), OLA (10 mg daily, NA) pregabalin (100 mg daily, NA) b) ARI (30 mg daily, NA), lithium carbonate (800 mg daily, NA) c) HALO-LAI (50 mg monthly, NA), HALO (10 mg daily per os, NA) d) ARI-LAI (400 mg monthly, NA)
Sweet et al. (86)	Case report (USA)	1, 47, African-American, M	None	SC	Bizarre behavior, delusions, hallucinations, agitation, aggressiveness	NA	OLA (10 mg daily, NA), HALO (10 mg im, NA), LORA (2 mg, NA), dipgenhydramine (50 mg, NA)
Skryabin and Vinnikova (87)	Single-center analysis cohort study (Russia)	46, 23.2 ± 3.5 (mean), NA, M	NA	SC	Delirium, tactile hallucinations, auditory verbal hallucinations, Kandinsky–Clerambault's syndrome delusion of influence, automatisms, anxiety, paranoia, delusional mood, agitation, delusional persecutory ideas, thought insertion or withdrawal, etc.	Cannabis, alcohol	NA
**Studies on synthetic cathinone-induced psychosis**							
Antonowicz et al. (88)	Case report (USA)	a) 1, 27, NA, F b) 1, 32, NA, M	None	"Powdered Rush" containing MDPV	a) Tachycardia, diaphoresis, paranoid psychosis, disorganized though process, poor memory, insomnia b) Hypertension, tachycardia, disorganized behavior, paranoid psychosis, insomnia	Previous history of opiate dependence	a) Hospitalization, RIS (0.5 mg BID, NA) b) Hospitalization, refusal of any medications
Derungs et al. (89)	Case report (Germany)	1, 31, NA, M	NA	Naphyrone powder ingestion	Blurred vision, restlessness, anxiety, dysphoria, hallucinations, insomnia, paranoia	Regular MDMA, alcohol and benzodiazepine abuse	NA
Penders and Gestring (90)	Case report (USA)	3, NA, NA, 2 M and 1 F	None	MDPV	Hyperactivity, anger, confusion, hallucinations, paranoia, anxiety, fearful, insomnia	NA	Hospitalization, RIS (*n* = 2, 0.5 mg orally BID, NA), HALO (*n* = 1, 1 mg orally BID, NA)
Striebel and Pierre (91)	Case report (USA)	1, 22, NA, M	None	MDPV	Anxiety, hallucinations, chest pain, diaphoresis, nausea, tachycardia	Regular cannabis use	Hospitalization, supportive care, LOR (NA, NA)
Adebamiro and Perazella (92)	Case report (USA)	1, 26, NA, M	NA	Synthetic cathinones	Diaphoresis, kidney dysfunction, confusion, paranoia, agitation, hallucinations, hypertension, tachycardia	NA	Hospitalization
Kasick et al. (93)	Case report (USA)	a) 1, 38, Caucasian, M b) 1, 26, Caucasian, M	None	"Arctic Blast" and "Posh Aromatherapy Bath Salts" containing synthetic cathinones	a) Snakes hallucinations, aggressiveness, tachycardia, hyperthermia, agitation, alertness, anxiety, paranoia b) Auditory hallucinations, feelings of detachment, derealization, paranoia, suicidal ideation, confusion, delirium, tremors, hyperreflexia, myoclonus, amnestic effects	NA	a) Hospitalization, LOR (13 mg IV, NA) and supportive therapy ev, then HALO (5 mg IM, NA) b) Hospitalization, LOR (5 mg IV, NA), RIS (0.5 mg daily, NA)
Lajoie and Rich (94)	Case report (USA)	1, 50, NA, M	None	MDPV	Agitation, hallucinations, psychosis, suicidal ideation, chest pain, skin lacerations, tachycardia	History of methamphetamine abuse	Hospitalization, LOR (NA, NA), OLA (NA, NA)
Thornton et al. (95)	Case report USA)	1, 23, NA, M	Personal psychiatric history of psychotic disorder not better specified	MDPV	Bizarre behavior, suicidality, visual, tactile and auditory hallucinations, agitation, tachycardia, hyperthermia	NA	Hospitalization, LOR (6 mg daily, NA), droperidol (2.4 mg daily, NA)
Gunderson et al. (96)	Case report (USA)	1, 20, NA, M	Personal psychiatric history of MDD	"Infinity", "TranQuility," "Cool Wave," "White Lightning" containing synthetic cathinones + diphenhydramine	Irritability, weight loss, insomnia, hallucinations "seeing different things, including people walking around his yard and house dressed all in white, having sex in his yard, chanting, etc."), suicidal ideation, paranoid psychosis, defensive homicidality	Previous cocaine, alcohol and opioid dependence in stable remission Previous cannabis and SC consumption	Hospitalization
Khan et al. (97)	Case report (USA)	1, 19, NA, F	None	"Ivory Wave" containing synthetic cathinones	Severe agitation, aggressive behavior, psychosis, poisoning delusion, auditory and visual hallucinations, vernal aggressiveness, labile mood, tachycardia, hypertension, diaphoresis, mydriasis	None	Hospitalization, LOR (NA, NA) and OLA (NA, NA)
Mangewala et al. (98)	Case report (USA)	1, 15, NA, M	None	MDPV	Agitation, paranoid psychosis, barricading himself in his home, aggressiveness towards health professionals, Capgras syndrome	Previous cannabis use	Hospitalization, LOR (0.5 mg BID, NA), OLA (7.5 mg daily, NA)
Stoica and Felthous (99)	Case report (USA)	1, 30, Caucasian, M	Personal psychiatric history of bipolar affective disorder and schizophrenia associated with a family psychiatric history of schizophrenia and bipolar disorder	Synthetic cathinones	Suicidal threats and gestures, auditory hallucinations, euphoria, increased energy level, insomnia, paranoid delusion ("he thought his neighbors were at his door, watching for him"), homicide ideation towards his neighbors	Previous heroin, cannabinoids, BDZs, LSD, and methamphetamine abuse	Hospitalization, VA (250 mg BID, NA), trazodone (100 mg daily, NA)
Winder et al. (100)	Case report (USA)	1, 33, NA, M	None	MDPV and mephedrone	Diaphoresis, hyperthermia, tachycardia, agitation, dysphoria, euphoria, hallucinations, insomnia, paranoia	Prior methamphetamine, opioids, alcohol, benzodiazepines abuse	Hospitalization, LOR (NA, NA), QUE (NA, NA), unspecified AD
Bertol et al. (101)	Case report (Italy)	1, 27, Caucasian, M	Personal psychiatric history of bipolar disorder	MDPV ev ± 3-methyl-methcathinone ± pentedrone	Mydriasis, unresponsiveness, agitation, delirium, visual, tactile and auditory hallucinations, persistent sleeplessness, euphoria, micro-zoopsia	Previous alcohol dependence	Hospitalization, diazepam (NA), chlorpromazine (NA), ARI (NA)
Stevenson et al. (102)	Case report (UK)	1, NA, NA, M	Personal psychiatric history of drug-induced psychosis	3-MeO-PCP and MDPV	Vivid hallucinations, bizarre ideas, violence and aggressiveness, murder attempt	History of regular recreational use of several illicit drugs	NA
Crespi (103)	Case report (USA)	1, 17, NA, F	None	"Flakka" (Alpha-PVP)	Altered mental status, agitation, psychotic behaviors, cognitive deterioration, bizarre behavior	NA	Hospitalization, LOR (NA, NA) and OLA (10 mg BID, NA)
Dolengevich-Segal et al. (104)	Case report (Spain)	1, 25, NA, M	Personal psychiatric history of ADHD, antisocial behavior and early substance abuse in adolescence	Mephedrone in the context of ChemSex	Delusional paranoid ideation with an intense emotional and behavioral impact, visual hallucinations involving human forms and cellphone lights, suicide ideation, psychomotor restlessness, feeling of being "controlled," suspiciousness, cenesthopathy (i.e., "insects crawling under the skin")	Cocaine, alcohol, ketamine, GHB, MDMA, methamphetamine	Hospitalization, PALI (6 mg daily, NA) zonisamide (300 mg daily, NA), pregabalin (75 mg daily, NA)
Romanek et al. (105)	Retrospective single-center study (Germany)	81, 17–49, NA, 64% M	NA	Synthetic cathinones various	10 patients manifested prolonged psychosis	NA	NA
Richman et al. (106)	Case report (USA)	1, 20, North African descent immigrated to USA, M	None	"Flakka"	Anger, mystic delusions "believing to be the embodiment of a prophet"), aggressiveness, impulsivity, hyper-religiosity, command auditory hallucinations, visual hallucinations of serpents, suicide and homicidal ideation, mood lability, disorganized behavior, echopraxia, echolalia, stupor, staring	NA	Hospitalization, LOR (2–3 mg TID, NA), then clonazepam (2 mg BID, NA), ARI (10–25 mg daily, NA), then QUE (800 mg daily, NA)
Simonato et al. (107)	Case report (Italy)	1, 28, Caucasian, M	Personal psychiatric history of cannabis and skunk-induced psychotic episodes	"Flakka"	Depressive symptoms, lack of energy, visual hallucinations, panic attack, hyperthermia, sexual arousal, insomnia, Ekbom syndrome, persecutory delusion	Previous cannabis and skunk consume	Hospitalization, BUP (150 mg daily, NA)

NA, not available data; SC, synthetic cannabinoids; M, male, F female; BDZ, benzodiazepine; ED, emergency department; LOR, lorazepam, ev endovenous; ADHD, attention/deficit hyperactivity disorder; MDD, major depressive disorder; AC, anticonvulsants; AP, antipsychotics; HALO, haloperidol; RIS, risperidone; PTSD, post-traumatic stress disorder; ARI, aripiprazole; BUP, bupropion; OLA, olanzapine; ECT, electroconvulsant therapy; CLO, clozapine; VA, valproic acid; ICU, intensive care unit; QUE, quetiapine; ARI-LAI, aripiprazole long-acting injection; OR, odds ratio; PANSS, Positive and Negative Schizophrenia Scale; RIS-LAI, risperidone long-acting injection; HALO-LAI, haloperidol long-acting injection; 3-MeO-PCP, 3-methoxyphencyclidine; MDPV, methylene-dioxy-pyrovalerone; PALI, paliperidone.

## Results

### Data on NPS-Induced Psychosis in General

A cross-sectional survey carried out as a retrospective review of electronic discharge letters of all patients discharged from general adult psychiatric wards in the Royal Edinburgh Hospital recruited 483 admissions, 86 of which had NPS involvement reported. Among NPS users, the diagnosis of drug-induced psychosis was significantly higher (*p* < .001, OR = 18.7) compared to non-NPS users (25). The European Drug Emergencies Network (Euro-DEN) collected data on presentations to emergency department (ED) with acute recreational drug and/or NPS intoxication in 16 sentinel centers in 10 European countries, reporting psychosis varying from 3% to 16.3% (26). A retrospective analysis of cases presenting to the ED of the University Hospital of Bern, Switzerland, with symptoms/signs consistent with acute toxicity of recreational and/or NPS use identified two intoxications with NPS: one with methylone and one with 2C-P; 71 cases of psychoses even though the authors do not clearly describe clinical presentation related to the NPS identified (27).

### Data on Synthetic Cannabinoids

A case series described some instances of SC-induced reemergence of florid psychotic symptomatology among five forensic patients who took a mixture of SC (like “K2” and JWH-018) contained in “Aroma” blend (28). Psychopathological patterns comprised the onset of psychomotor agitation, disorganized behavior, and ideation, delusional beliefs (grandiose and paranoid) in previously stable patients with a personal history of severe mental illness (SMI) (28). No data are provided about pharmacotherapy and/or specific/suggested treatment (28).

Müller et al. (29) described a 25-year-old man, with a previous history of recurrent psychotic episodes triggered by cannabis consumption and stable on monotherapy with amisulpride (800 mg daily), who presented to hospital with increased levels of anxiety, feelings of manipulation, and psychotic symptoms after smoking “spice” drugs on three different occasions (3 g each). The subject reported the feeling of being controlled and manipulated through a chip which he thought was implanted in his abdomen several years before together with the onset of imperative voices and paranoid hallucinations, symptoms he never had before (29). no data are provided about pharmacotherapy and/or specific/suggested treatment (29).

Another case report described three cases of “Mojo psychosis,” a mixture of SC (30). No further details on psychopathological presentation have been reported by the authors (30).

A study evaluating urine metabolism of SC described three cases of new-onset psychosis following the intake of a mixture blend named “Tropical synergy” containing several SC (31). No further data are provided on psychopathological pattern and/or about pharmacotherapy and/or specific/suggested treatment (31).

Vearrier and Osterhoudt (32) described a case of an adolescent girl who arrived at the ED agitated, violent, and uncontrollable after inhaling a “K2.” She described a previous history of alcohol and cannabis intake. Her psychopathological pattern comprised the onset of visual hallucinations, anxiety, restlessness, tachycardia, higher blood pressure, muscle fasciculation, hypokalemia, and mydriasis. She was given lorazepam 2 mg intravenously, with a good remission (32).

Benford and Caplan (33) reported a case of a 20-year-old honor college student who presented to the ED with severe anxiety/paranoia and auditory/visual hallucinations after smoking “Spice” drugs. Psychopathological pattern comprised the onset of increasing levels of anxiety, paranoia, and both auditory and visual hallucinations. The subject refused voluntary psychiatric admission. No data are provided about pharmacotherapy and/or specific/suggested treatment (33).

A case series described 11 Hispanic adolescents admitted to the South Miami Hospital Addiction Treatment Center in Miami-Dade County, Florida, after smoking SC and developing a new-onset psychosis (34). Subjects reported using Spice drugs more than three times in their lifetime (8 out of 11 subjects), while 4 out of 11 reported smoking SC multiple times per day. Psychopathological patterns comprised mood fluctuations, altered cognition and perception (i.e., visual and auditory), memory difficulty, euphoria, sometimes irritability and anxiety, and paranoid thoughts (34). In addition, subjects reported tachycardia, restlessness, appetite changes, muscle fasciculation, tremors, and weakness. No data are provided about pharmacotherapy and/or specific/suggested treatment (34).

A cohort explorative study recruiting subjects from a Regional Forensic and Rehabilitation service in New Zealand reported a SC-induced psychotic recrudescence in 15 vulnerable individuals previously affected with SMI (35). The psychopathological patterns comprised the onset of psychomotor agitation, disorganized behavior, and ideation, delusions after smoking SC. All subjects reported a previous psychiatric history of psychotic disorder and had been compulsorily treated with therapeutic doses of antipsychotics (unspecified) and, in five cases, together with mood stabilizers (unspecified). No data are provided about pharmacotherapy and/or specific/suggested treatment following SC-induced psychosis (35).

Forrester et al. (36) retrospectively collected data on SC exposures referred to Texas Poison Centers in 2010 by identifying new-onset psychoses following SC intake. Psychopathological patterns comprised the onset of psychomotor agitation and hallucinations. In addition, tachycardia, hypertension, nausea, vomiting, drowsiness, and multiple neurological symptoms were reported. No further information is provided regarding the psychopathology and phenomenology of SC-induced psychoses. No clear data are provided about specific/suggested treatment following SC-induced psychosis, intravenous fluids, benzodiazepines, oxygen, and antiemetics being reported, among the most commonly prescribed medications and, in around 3% of subjects, the use of sedatives and anticonvulsants (36).

Hurst et al. (37) described 10 cases of otherwise healthy men, admitted to the psychiatry ward at the San Diego Naval Medical Center between August and December 2010, who experienced a SC-induced new-onset psychosis. The psychopathological patterns were characterized by auditory and visual hallucinations, disorganized behavior and speech, suicidal ideation, alogia, insomnia, psychomotor agitation and/or retardation, and higher levels of anxiety. No clear data are provided about specific/suggested treatment following SC-induced psychosis, use of antipsychotics (unspecified) being reported in 10 patients (36).

A case report of a 23-year-old high-functioning Caucasian Navy corpsman who developed a SC-induced psychosis was reported by Johnson et al. (38). The psychopathological pattern comprised the onset of nonsensical speech, paranoia of being videotaped, disorganized behavior and speech, tangential thoughts, delusional ideation (i.e., the feeling of own “mind expanded” and the ability to “comprehend infinity,” and so on) without perceptual distortions. The psychotic symptoms of the subject spontaneously resolved after abstinence from SC about 24 h after initial presentation. No data are provided about psychopharmacology and/or specific treatment of SC-induced psychosis (38).

Data collected retrospectively by the Italian database of the Pavia Poison Centre reported 17 cases of SC users who exhibited various psychiatric and clinical patterns, including tachycardia, agitation/anxiety, confusion, hallucinations, mydriasis, paresthesia, palpitations, drowsiness, xerostomia, syncope, seizures, vertigo, tremor, hypertonia, coma, etc. (39). The authors reported symptomatic treatment and benzodiazepines for treating neuroexcitatory effects (39). No further details regarding psychopathological pattern of SC-induced psychosis were reported by Locatelli et al. (39).

McCain et al. (40) retrospectively described six cases of SC-induced psychopathological/clinical pattern referred to the Arkansas Poison and Drug Information Center. Psychopathological patterns comprised agitation, irritability, and hallucinations. Benzodiazepines, intravenous fluids, antiemetics, and potassium supplementation were prescribed, among treatments, without details about dosage(s) and/or treatment duration (40).

Simmons et al. (41) described three cases of young adults referred to an ED after “Spice” intake (JWH-018 and JWH-073) who exhibited anticholinergic and sympathomimetic clinical effects and a new-onset SC-induced psychosis. Benzodiazepines (intravenous lorazepam, 4 mg), antipsychotics (haloperidol, 5 mg), supportive therapy, and observation were among the treatments prescribed (41).

A 17-year-old adolescent boy without significant previous psychiatric and medical history presented to an ED reporting “pounding in his chest,” constant chest pressure, and a new-onset SC-induced psychosis after smoking a SC, called K9 (42). No data are provided about pharmacotherapy and/or specific/suggested treatment (42).

Other case reports of SC-induced new-onset psychoses have been reported by McGuinness and Newell (43). Psychopathological patterns comprised aggressiveness, paranoia, short-term memory deficits, and increasing levels of anxiety. No data are provided about pharmacotherapy and/or specific/suggested treatment (43).

Tung et al. (44) described a 36-year-old male real-estate agent with a previous polysubstance abuse (heroin, codeine, and cannabis) with a family psychiatric history of SMI (unspecified) and substance use disorder (SUD) who presented an episode of acute psychotic disorder characterized by florid persecutory delusion, auditory hallucinations, disorganized behavior, irritability, aggressiveness, and agitation. No further data are provided about pharmacotherapy and/or specific/suggested treatment (44).

Van der Veer and Friday (45) described three patients who presented with severe, persisting psychotic symptoms after regular SC use for 3–4 weeks prior to admission. Psychopathological patterns comprised disorganized speech and behavior, paranoia, bizarre delusions, suicidality, aggressiveness, poverty of thoughts, loosening of associations, poor attention/concentration, and inappropriate affect; onset of Capgras delusions was reported in one case. Two out of three patients did not have a previous psychiatric history, while one reported a post-traumatic stress disorder (PTSD). All three patients required hospitalization and were treated with antipsychotic medications (haloperidol in two cases and risperidone in one case). No further data are provided regarding dosage(s) and/or treatment duration (45).

Bebarta et al. (46) described three cases of “Spice” use in military members followed by onset of psychotic symptomatology. Psychopathological patterns comprised the onset of paranoia, aggressiveness, agitation, and visual hallucinations. Regarding the treatment, intravenous lorazepam 2 mg was reported for managing agitation, with resolution of symptomatology (46).

Cohen et al. (47) reported three cases of adolescents who presented with a new-onset SC-induced psychosis characterized by catatonia, psychomotor agitation, and aggressiveness. No further data are provided about pharmacotherapy and/or specific/suggested treatment (47).

A retrospective series of exposures to SC coming from the US National Poison Data System between January and October 2010 were analyzed by Hoyte et al. (48) by identifying new-onset psychosis induced by SC products. No further data are provided either regarding further specific psychopharmacological treatment beyond benzodiazepines or about psychopathological patterns of SC-induced psychosis (48).

Two adolescents who manifested a new-onset SC-induced psychosis have been reported by Oluwabusi et al. (49). Both patients reported a family psychiatric history for schizophrenia and/or bipolar disorder. Psychopathological pattern comprised disorganized behavior, paranoid delusions, insomnia, hyperactivity, anxiety, musical auditory hallucinations, mood lability, irritability, euphoria, pressure of speech, flights of ideas, paranoia, and grandiose delusions. Regarding treatment, one subject was firstly stabilized on quetiapine and then changed to aripiprazole (20 mg daily) after the onset of an acute dystonic reaction, while the other one was prescribed olanzapine (15 mg daily bedtime) (49).

Another case report has been described of a new-onset SC-induced psychosis in a 59-year-old Latino male with a history of PTSD and polysubstance abuse (heroin, cocaine, cannabis, and alcohol) (50). His psychopathological pattern comprised visual hallucinations and disorganized and bizarre behavior. He was treated with benzodiazepines, gabapentin (400 mg QID), hydroxyzine (25 mg TID), aripiprazole (10 mg qD), benztropine (1 mg BID), and bupropion (150 mg BID) (50).

An Internet-based survey reported the onset of psychotic symptomatology in 28% of a sample (*n* = 168) of SC users recruited from 13 different countries (51), while an Australian online survey (*n* = 316) reported paranoia (18%) and psychosis (4%) (52). No further data are provided about pharmacotherapy and/or specific/suggested treatment in both surveys (51, 52).

Berry-Cabán et al. (53) described a 20-year-old Hispanic male who presented to an ED due to an altered mental status and delirium following SC intake. Initially, the subject was uncommunicative, awake then non-compliant, combative, and aggressive; hence, clinicians applied physical restraint and firstly administered 2 mg/ml parental lorazepam for managing increasing levels of agitation. During hospitalization, he was additionally treated with further 4 mg of lorazepam, 5 mg of haloperidol, and 25 mg of diphenhydramine. His psychopathological pattern comprised agitation, aggressiveness, disorganized speech and behavior, hallucinations, and paranoid ideation. He was discharged with a prescription of risperidone 1 mg daily. No further data on treatment duration has been provided (53).

Chan et al. (54) described a 21-year-old man without a previous psychiatric history who presented to an ED due to the onset of increasing levels of psychomotor agitation and paranoid thoughts after consuming 0.4 g of 6-APB and smoking cannabis over a 2-day period. Psychopathological pattern comprised agitation, paranoia, the belief that others could read his mind, self-harm, and suicidality. He was treated with diazepam (4–10 mg daily) for managing agitation during hospitalization. No further data have been provided regarding further treatments to be prescribed and/or treatment duration (54).

A retrospective cohort study targeting individuals seeking emergency treatment after SC intake and selected from the database of the Freiburg Poisons Information Center between September 2008 and February 2011 reported a plethora of physical symptoms and psychotic presentation (55). Psychopathological patterns included visual and auditory hallucinations, confusion, agitation, delirium, restlessness, and psychotic symptoms. Supportive care, talk down strategies, intravenous fluids, potassium supplementation, and benzodiazepines were prescribed. No further data have been provided regarding specific psychopharmacotherapy and/or treatment duration and/or dosage(s) (55).

A case report of an Italian male who consumed "Bonzai," a mixture of SC, has been presented by Papanti et al. (7). The subject did not have any psychiatric history and his psychopathological pattern was characterized by increasing levels of anxiety, insomnia, ideas of reference, somatic and visual hallucinations, autoscopy, and aggressiveness. He was initially treated with diazepam 5 mg intravenously and was rehydrated, and then he was prescribed olanzapine 5 mg daily and bromazepam 3 mg daily for 4 weeks (7).

Glue et al. (56) retrospectively collected data on 17 patients hospitalized in an acute psychiatric ward, located in New Zealand, following the use of a SC (“K2”) and described their clinical and psychopathological features. Nine out of 17 patients had recurrences of preexisting disorders and four presented with new psychotic symptoms. Psychopathological patterns comprised paranoia, thought disorder, disorganized behavior and speech, anxiety, mood lability, and intense suicidal thinking and/or behavior. Antidepressants and antipsychotics (unspecified) were prescribed, without specifying treatment duration and/or dosage(s) (56).

Leibu et al. (57) described a case of a 36-year-old African-American man with a previous long-lasting psychiatric history of schizophrenia who presented with severe and life-threatening catatonia after consuming SC. Catatonia was successfully treated with electroconvulsive therapy, while psychotic symptoms have been managed with 225 mg BID clozapine (57). No further data are available regarding treatment duration (57).

A Turkish survey carried out on a sample of 158 patients who were admitted to Bakirkoy Research and Training Hospital for Psychiatry, Neurology and Neurosurgery, Alcohol and Drug Research, Treatment and Training Center (AMATEM) revealed a new onset of hallucinations and delusions among SC consumers (58). Psychopathological patterns comprised increasing levels of anxiety (75.6%), irritability (18.6%), insomnia (61.5%), hallucinations (40.4%), and delusions (16%). No data have been provided regarding specific psychopharmacological treatment of SC-induced psychosis (58).

A case series of four paranoid schizophrenic patients who took SC showed a worsening of psychotic symptomatology (59). All subjects were hospitalized and were previously prescribed specific medications for their psychiatric disorder (i.e., haloperidol decanoate, quetiapine, risperidone, diazepam, clozapine, lorazepam, olanzapine, clonazepam, and fluoxetine). Psychopathological patterns comprised cognitive deficits, anhedonia, dysphoria, avolition, blunted affect, incoherent speech, thought blocking, severe agitation, increasing levels of anxiety, and so on. The subjects were furtherly treated by increasing benzodiazepine dosages (lorazepam and/or diazepam) (59).

Haro et al. (60) described a 19-year-old Hispanic woman who developed a psychotic episode after consuming SC. Her psychopathological pattern comprised self-references when eating or walking, visual hallucinations, soliloquy, laughter forfeit, catatonia, depersonalization, and derealization. After 2 months of abstinence from SC and treatment with aripiprazole (15 mg daily), lorazepam, and biperiden, psychotic symptomatology partially resolved (60).

Meijer et al. (61) described a case of bilateral upper-extremity self-mutilation and new-onset SC-induced psychosis in a healthy 26-year-old man presented to an ED, with a previous history of attention deficit hyperactivity disorder (ADHD), treated with lisdexamfetamine dimesylate. His psychopathological pattern comprised the onset of paranoid delusions causing him to feel his hands were going to harm him, then he placed them on the stove and attempted to burn them off to “get the devil out of him.” No further data have been provided regarding the psychopharmacological treatment following the discharge (61).

A 17-year-old male who developed catatonia and new-onset SC-induced psychosis was reported by Smith and Roberts (62). His psychopathological pattern comprised confusion, bizarre behavior, mutism, rigidity, excitatory catatonia, auditory and visual hallucinations, and disorganized thoughts. Lorazepam 2 mg intramuscular was administered for managing agitation, followed by six sessions of electroconvulsive therapy and olanzapine. No further data have been provided regarding dosage(s) and/or treatment duration (62).

Durand et al. (63) described a case of new-onset SC-induced psychosis associated with severe rhabdomyolysis after consuming SC in a 23-year-old man without prior psychiatric history. His psychopathological pattern comprised altered mental status, severe agitation, acute psychosis, irritability, persecutory delusions, and impulsiveness. Intravenous lorazepam every 6 h was prescribed for managing agitation, then haloperidol 10 mg TID, valproic acid 500–1,000 mg, and lorazepam 2 mg TID. No further data on treatment duration has been provided (63).

Schwartz et al. (64) reported seven cases who presented simultaneously at a hospital in Georgia (USA), after smoking a mixture of SC contained in a blend named “Crazy Clown” during a party. Psychopathological patterns comprised a new onset of SC-induced psychosis, aggressiveness, extreme agitation, and increasing levels of anxiety. Regarding the treatment, two patients were admitted to the intensive care unit due to persistent hypertension and tachycardia and mental status alterations and were intubated. One patient presented with cardiac arrest and was resuscitated by paramedics and underwent successful balloon angioplasty; the other subjects were not treated due to non-compliance and/or spontaneous remission of symptomatology. No further data have been provided regarding specific pharmacological treatment (64).

Ustundag et al. (65) described a case of SC-induced mania with psychotic symptoms in an 18-year-old single boy who developed an increasing speech, spending money, a great deal of interest in religion and insomnia, mystic delusions, irritability, euphoria, mood lability, and without hallucinations after consuming SC. Olanzapine (from 10 to 20 mg daily), valproic acid (from 500 to 1,500 mg daily), quetiapine (from 200 to 400 mg daily), and lorazepam (from 0,5 to 1 mg daily) were prescribed. No further data on treatment duration are available (65).

Sönmez and Köşger (66) described a 31-year-old man who developed a new-onset SC-induced psychosis after consuming a mixture of SC called "Bonsai" three times a week for more than 6 months and was treated with olanzapine.

Rahmani et al. (67) described two cases with a strong family psychiatric history who developed a SC-induced psychosis following the intake of “Spice.” Psychopathological pattern comprised the onset of paranoia, bizarre and disorganized behavior and speech, and visual hallucinations. No further data have been provided regarding treatment duration and/or dosage(s) of clozapine prescribed in both cases (67).

An observational case series (*n* = 35) retrospectively collected laboratory analysis of patients presenting to an ED with a documented suspicion of SC intake and described a new onset of SC-induced psychosis (68). Psychopathological patterns comprised altered mental status (61%), hallucinations (6%), and seizures (40%). Five patients were ventilated and intubated. No further data on specific psychopharmacological treatment has been provided (68).

A cross-sectional study recruiting 81 male patients diagnosed with SC-induced psychotic disorder (*n* = 50) or with schizophrenia (*n* = 31) who were concurrently hospitalized described a higher rate of suicidal ideation, involuntary hospitalization, as well as similar clinical picture with schizophrenia by inducing paranoia, disorganized behavior, visual and auditory hallucinations, and suicidal thoughts, not only in vulnerable subjects but also in subjects without a previous history of psychosis (69). Furthermore, verbal learning, short-term memory and working memory, executive functions, abstract ability, and decision-making and attention functions were reported to have been impaired among SC-induced psychotic subjects (69).

Khan et al. (70) described two cases of SC-induced catatonic state associated with psychosis, psychomotor alterations, speech and behavior disorganization, flatted affect, alogia in a 17-year-old male with no psychiatric history who presented to the ED with psychosis after a 2-week Spice binge and in a 21-year-old male who reported a history of childhood ADHD with progressive isolation and negative symptoms after heavy SC consumption. The first case was treated with aripiprazole 7.5 mg daily and lorazepam (2 mg BID), then olanzapine (unspecified dosage) and valproic acid (up to 100 mg qHS) were added while aripiprazole was tapered off, while the second case was treated with lorazepam for managing catatonia and firstly risperidone (1–2 mg BID), then switched to aripiprazole (5 mg daily, titrated up to 25 mg daily) (70).

A retrospective observational case series of patients (*n* = 22) presenting to an ED with analytically confirmed SC intake were described from a pharmacological, toxicological, and clinical point of view by Hermanns-Clausen et al. (71). Psychopathological patterns comprised restlessness, agitation, and visual hallucinations. Treatment was mainly supportive, benzodiazepines (unspecified), metoprolol, and antiemetics. No further data have been provided regarding dosage(s) and treatment duration (71).

A retrospective chart study (*n* = 594) evaluating all patients who were admitted to a dual diagnosis psychiatric unit at Mount Sinai Beth Israel in New York City compared SC users vs. cannabis users by reporting more psychotic symptoms (*p* = .012) and more agitation (*p* < .001) among SC consumers (72). Among medications, more antipsychotics alone (31.4% vs. 19.6%) or a combination antipsychotic plus mood stabilizers/antidepressants (57.1% vs. 33.5%) were prescribed among SC-only users compared to cannabis-only users (72).

A case report described an 18-year-old antipsychotic-naive African-American male without past psychiatric history admitted after presenting to the psychiatric emergency room with a first-time psychotic episode after a prolonged SC ingestion *via* inhalation (73). The subject was treated with intermittent doses of lorazepam 2 mg orally and stabilized over 1.5 weeks on oral risperidone (5 mg daily) (73).

An 18-year-old Hispanic male admitted to the ED after 5 days of acute-onset auditory hallucinations, paranoid delusions, and panic attacks with a previous history of cannabis abuse and more recently of SC, purchased from Internet blog websites, has been described by Samaan et al. (74). No further data about specific psychopharmacological treatment of SC-induced psychosis has been provided (74).

Ozer et al. (75) described a case of an adolescent with a new-onset psychosis following inhalation of a mixture of SC called “Bonsai.” Olanzapine (10 mg daily) was prescribed and the subject was followed up 3 months after discharge without a recurrence of psychotic symptoms (75).

A cohort study reported, among SC consumers, higher psychotic levels compared to cannabis users (76). No further data about specific psychopharmacological treatment of SC-induced psychosis has been provided (76).

A retrospective cohort study recruiting data from the UK National Poisons Information Service reported hallucinations and paranoia as consequences of SC intake (77). No further data about specific psychopharmacological treatment of SC-induced psychosis has been provided (77).

A case–control neuroimaging study comparing SC users vs. healthy controls, using magnetic resonance imaging, reported that chronic SC use was associated with dose-dependent downregulation of CB_1_ receptors, lower fractional anisotropy (FA) values in the left inferior fronto-occipital fasciculus (IFOF), and inferior longitudinal fasciculus (ILF) in the left temporal lobe which might be associated with increased risk of the development of psychosis (78). No further data about specific psychopharmacological treatment of SC-induced psychosis has been provided (78).

A case report described a patient who developed acute psychosis, aggressiveness (e.g., homicidal and violent behavior towards staff), and new-onset refractory status epilepticus necessitating emergent neurological life support and prolonged admission to an intensive care unit following SC consumption (79). The patient was treated with intravenous lorazepam, valproic acid, levetiracetam, and lacosamide. Following a 3-week hospitalization in the intensive care unit, the patient was discharged and followed up at 1 month. No further data on dosage(s) and/or treatment duration are available (79).

A cohort study systematically described the clinical characteristics of 100 SC users in a large sample from an urban public hospital in New York City by reporting a high rate of acute psychotic symptoms, particularly among the already socially vulnerable and psychiatrically ill population of the sample (80). Most SC users (73.7%) were prescribed an antipsychotic medication on discharge. No further data on dosage(s) and treatment duration (80).

Monte et al. (81) collected data from the US ToxIC (2010–2015) and found that among 353 SC consumers, about 40% had SC-induced delirium and toxic psychosis.

A qualitative study of six SC users in Hungary revealed the onset of paranoia and a synthetic psychosis among participants (82). Similarly, an online survey reported the onset of agitation, paranoia, and other psychotic symptomatology following SC intake (83). No further data are available regarding specific psychopharmacological treatment of SC-induced psychosis in both studies (82, 83).

A prospective pilot study recruiting 332 patients with cannabis and/or SC use evaluated the psychosis-inducing potential of cannabis vs. SC, reporting more severe psychotic symptoms among SC vs. cannabis users (84). No further data about specific psychopharmacological treatment of SC-induced psychosis has been provided (84).

A case series described four patients taking SC and presenting to a UK acute psychiatric unit with a SC-induced psychosis by reporting mixed hallucinations, persecutory delusions and thought disorganization, physical and verbal aggressiveness, and sexual disinhibition (85). All subjects recruited were treated with benzodiazepines and antipsychotic drugs, as shown in **Table 2**.

A 47-year-old African-American man presented for involuntary inpatient psychiatric admission after being brought in by police secondary to bizarre behavior/hallucinations/agitation/delusions following SC intake (86). He was initially treated with olanzapine (10 mg daily), then switched to haloperidol intramuscularly and lorazepam due to aggressiveness and increasing levels of agitation towards clinicians. The patient was observed and he refused any follow-up and/or medication after discharge (86).

A single-center cohort study evaluating hospitalized SC-induced psychotic patients in a Russian hospital identified specific clinical variants of psychoses among SC users (87). No further data about specific psychopharmacological treatment of SC-induced psychosis has been provided (87).

### Data on Synthetic Cathinones

Antonowicz et al. (88) described two cases of paranoid synthetic psychosis in a 27-year-old female and in a 32-year-old male who consumed MDPV. Psychopathological patterns comprised the onset of paranoid ideation, disorganized thought process, insomnia, and agitation. The first subject was successfully treated with low doses of risperidone, while the second case refused any medications (88).

Penders and Gestring (90) described the onset of a hallucinatory delirium following use of MDPV in three cases who presented to an ED of a tertiary care hospital in the USA. Two cases were successfully managed with risperidone (0.5 mg orally BID), while the third one was treated with haloperidol (1 mg orally BID) (90).

Striebel and Pierre (91) described a 22-year-old man without any previous psychiatric history who used cannabis regularly to manage his Crohn's disease and who developed a new-onset MDPV-induced psychosis. His psychopathological pattern comprised the onset of hallucinations, altered perceptions, “seeing things move that shouldn't be moving,” feeling an earthquake, and agitation. He was treated with lorazepam and supportive care (91).

Lajoie and Rich (94) described a 50-year-old male with a previous history of methamphetamine dependence who developed a MDPV-induced psychosis characterized by self-mutilation, suicidality, agitation, increasing levels of panic attack, and auditory hallucinations. He required sedation with olanzapine and lorazepam, for managing agitation. No further data on dosage(s) and treatment duration have been provided (94).

Thornton et al. (95) reported a 23-year-old man with a previous psychiatric history who presented to an ED after developing a bizarre and disorganized behavior, suicidality, visual, tactile, and auditory hallucinations, and agitation following the intake of MDPV. He was treated with 6 mg of lorazepam and 2.4 mg of droperidol intravenously over 90 min in order to manage the agitation. He was also taking aripiprazole, valproic acid, lithium, quetiapine, and clonazepam for his psychiatric disorder. No further data on treatment duration and/or dosage(s) are available (95).

Winder et al. (100) described MDPV-induced paranoid psychosis in a 33-year-old male veteran without a previous psychiatric history. He was treated with as-needed doses of quetiapine and lorazepam for paranoid ideation, agitation, and anxiety. Symptomatology resolved within 12 h after admission. He was then started on an antidepressant (unspecified) for residual symptoms of depressed mood, anhedonia, and hopelessness. No further data on treatment duration and/or specific psychopharmacological treatment for MDPV-induced psychosis are available (100).

Bertol et al. (101) described a case of mixed MDPV and benzodiazepine intoxication in a 27-year-old chronic abuser with a previous SUD and a psychiatric history who developed severe agitation, disorganized and bizarre behavior, suicidality, and visual, tactile, and auditory hallucinations. He was managed with diazepam intravenously due to agitation and then he started chlorpromazine due to sleeplessness and aripiprazole for managing psychotic symptoms. No further data on treatment duration and/or dosage(s) are available (101).

Mangewala et al. (98) reported a psychotic onset (i.e., delirium, agitation, paranoia, and hallucinations) in a 16-year-old adolescent man without a previous personal and/or family psychiatric history who abused synthetic cathinones. He was treated with a combination of olanzapine (from 5 to 7.5 mg daily) and lorazepam (0.5 mg BID) (98).

Derungs et al. (89) reported a case of paranoia and psychosis induced by Naphyrone in a 31-year-old man. Psychopathological pattern comprised agitation, restlessness, insomnia, anxiety, and hallucinations. No further details have been provided regarding a specific psychopharmacological treatment of synthetic psychosis (89).

Two cases of severe intoxication delirium with paranoid psychosis and hallucinations following “bath salts” consumption are described by Kasick et al. (93).

Khan et al. (97) described a case of a 19-year-old female, without a family and/or personal psychiatric history, who developed a paranoid psychosis after consuming synthetic cathinones. She was treated with olanzapine and lorazepam. No further data on dosage(s) and/or treatment duration have been provided (97).

Adebamiro and Perazella (92) reported a case of a new-onset psychosis associated with renal and cardiovascular dysfunction following “bath salts” intoxication. The subject was treated with supportive care and hospitalized (92).

Gunderson et al. (96) described a case of paranoid psychosis induced by “bath salts” and diphenhydramine in a subject with a previous psychiatric history of major depressive disorder (MDD).

Stoica and Felthous (99) described a case of acute psychosis in a 30-year-old subject with a psychiatric diagnosis of bipolar affective disorder and schizophrenia following the intake of synthetic cathinones. Psychopathological pattern comprised suicidality, homicidal tendencies, euphoria, alertness, paranoid delusion, and increased levels of energy. The subject was hospitalized and started initially on valproic acid (250 mg orally BID) and trazodone (100 mg daily at night) for managing impulse dyscontrol and insomnia. No further data are available on treatment duration (99).

A case report described a psychotic onset, associated with the attempted murder of his father, in a man with a history of regular recreational use of a wide range of illicit drugs between 14 and 20 years following the intake of 3-methoxyphencyclidine (3-MeO-PCP) and MDPV (102). The patient experienced vivid hallucinations (auditory, visual, and tactile), panoramic visual hallucinations (“full brown eyes closed visuals” and imperative voices saying “kill your father”), and bizarre ideas (e.g., “using house as a base for super heroes”), without other symptoms of schizophrenia such as delusional beliefs or thoughts disorder (102).

Several case reports of alpha-PVP (aka “Flakka”)-induced psychosis have been described (103, 106, 107). Crespi et al. (103) described a 17-year-old who developed a Flakka-induced prolonged psychosis characterized by altered mental status, agitation, auditory hallucinations, disorganized behavior, and psychotic symptoms. She was managed with olanzapine and lorazepam (103). A 20-year-old male who ingested one tablet of Flakka developed a psychosis characterized by agitation, aggressiveness, hyper-religiosity, command auditory and visual hallucinations of serpents, suicidality, homicidal tendencies, and delusions. He was initially managed with 2 mg of lorazepam intramuscularly for agitation, then he was prescribed oral lorazepam 2 mg TID and aripiprazole 10 mg daily for 3 weeks, then titrated lorazepam 3 mg TID and aripiprazole 24 mg daily. After modest improvement, he was switched from lorazepam to clonazepam (2 mg BID) only for 3 days and then restarted on lorazepam, while aripiprazole was modified with quetiapine (800 mg daily), with marked improvement of psychotic symptoms and catatonia (106). Simonato et al. (107) reported a case of “Flakka-induced” psychosis successfully treated with bupropion.

A case of psychosis onset following the use of mephedrone in the context of ChemSex has been reported by Dolengevich-Segal et al. (104), as described in **Table 2**.

A cohort study reported the major complication of cathinone use as a prolonged psychosis, with a high proportion of cases among MDPV and methylone consumers (107). No further details have been provided regarding a specific psychopharmacological treatment of synthetic psychosis (105).

## Discussion

Overall, NPS use seems to exert stronger and more persistent and severe effects among subjects with SMI than in healthy subjects (i.e., without a previous psychiatric history), mostly due to activity in the dopaminergic system, implicated in managing behavior and thought processes and in determining psychosis, or in the serotonergic, noradrenergic, and/or glutamatergic systems (4, 108, 109) (as illustrated in **Table 1**). NPS may exert severe dissociative states, confusion in previously psychotic patients, relapse or worsening of a preexisting psychosis, persistent worsening of psychotic symptoms course, or the onset of a new severe psychotic symptomatology among healthy subjects (110, 111). In fact, the emergence of a psychotic symptomatology is commonly associated with a plethora of neurotransmission changes, e.g., increased central dopamine levels, cannabinoid CB_1_ receptor activation, 5-HT_2A_ receptor activation, decreased activity in *N*‐methyl‐‐aspartate receptors, and k‐opioid receptor activation. NPS may interfere at these neurobiological levels and facilitate the imbalance of several neurotransmitters and receptors (as illustrated in **Table 1**). For this reason, NPS use may really determine severe psychiatric symptoms, also in individuals not previously affected by a mental disorder. It is still unclear how the frequency of use (continuous vs. discontinued), the intensity of consumption (low, medium, or heavy), and the dosages (low vs. high dosages) may or not influence the development of a specific psychopathological pattern. Therefore, the advent of NPS has posed further clinical concern as not only are their clinical, toxicological and safety profiles often completely unknown (1) but they may also cause the onset of new psychopathological entities or the reemergence of “forgotten” psychopathological patterns, such as HPPD (4, 5).

Furthermore, the subtle gap/bridge between a “classical” vs. a “synthetic” psychosis is still the subject of clinical concern, as it seems the psychopathological, phenomenological, and clinical features of these two entities remain completely undefinable and clear. Moreover, this “gap” may have also meaningful effects in the choice of the best treatment and in defining the best outcome(s) and prognosis.

In addition, even though generically classified as NPS, not all NPS possess the same pharmacological and clinical profiles; hence, from a psychopathological point of view, clinicians may observe several types of NPS-induced “synthetic psychoses,” depending on the substance involved. Similarly, a specific NPS may cause different psychopathological effects in vulnerable vs. non-vulnerable individuals (as shown in **Table 2**). With regard to this, the model of a substance-related exogenous psychosis (SREP) and its toxic subtype (aka “lysergic psychoma”) may be helpful in shedding light to clinicians on differentiating classical/endogenous versus synthetic (NPS-induced) psychoses, from a clinical perspective. Unlike “classical/endogenous” psychosis, SREP is characterized by the following features: a) qualitative and quantitative consciousness alterations (i.e., crepuscular state and onyroid state); b) ego disorders (i.e., somatosensory/allopsychic); c) sensorial–perceptual disorders (i.e., visual, auditory, and coenesthetic); d) egodystonia (i.e., behaviors not coherent with self-image); e) mood swings; f) hyper-presentation of the time/“concrete” psychoses, i.e., alteration of the experienced space; g) modification of body perception; h) anhedonia/apathy/negative symptoms; and i) dyscontrol impulsivity and self-/hetero-aggressiveness (112) (as shown in **Table 3**). “Lysergic psychoma” is a phenomenological construct characterized by the perception of an “extraneous/foreign” body in one’s own mind, in which the residual critical ego takes position against the intoxicated part of one’s own self (113). It is a syndrome characterized by a clear egodystonic experience in which a subject clearly feels and observes this “foreign entity” as an unusual experience, out of own control, accompanied by hallucinations (mainly visual and kinaesthetic) and delusional perceptions (and thoughts) which are completely resisted by the subject who tries to stem them (114). This psychotic experience is often described as self-limiting, intense, and brief and appears to spontaneously resolve after substance discontinuation, as also observed in several studies examined here (see **Table 2**). However, chronic, persistent NPS use, mainly at high dosages, may cause a complex, persistent, and long-lasting psychopathological pattern, defined as “synthetic psychosis,” a paraphrenic syndrome due to a NPS-induced mental automatism which causes a psychotic trajectory (114). Furthermore, the pharmacodynamics and pharmacological profile of each NPS seem to be responsible for the psychopathological and clinical manifestation of each NPS-induced synthetic psychosis (as shown in **Tables 1**, **2**, and **4**) (1, 19, 115–117). Although there is still a need to clearly discriminate and characterize specific psychopathological and pathognomonic patterns depending on the specific NPS classes involved, as previously stated and shown in **Table 2**, an attempt had been made here to provide a critical summary of studies in order to describe some NPS-induced psychopathological clusters (**Table 4**).

**Table 3:** Classical psychosis vs. synthetic psychosis: psychopathological and phenomenological profile.

Endogenous psychosis (classical psychosis)	Exogenous psychosis (synthetic psychosis)
Lucid consciousness	Crepuscular consciousness
Thought disorders	Paraphrenia
Loss of contact with reality due to an ontological insecurity of the self	Loss of contact with reality due to an instability of the object ("floating world")
Primary, metaphysic, systematic delusion	Secondary, common, episodic delusion
Hallucinations	Pseudo-hallucinations
Transcendental ego	Empiric ego
Poor/absent insight, passivity	Present insight, activity
Bizarre and inexplicable behaviors	Aggressiveness and impulse dyscontrol
Apathy, anedonia, flattened affectivity	Overexcited, excessive affectivity

**Table 4:** Clinical variants of NPS-induced psychosis.

*Synthetic psychosis with predominant delirium symptoms* induced by NPS mainly acting on GABA, Ach, DA, SER, NA, and GLU pathways	Delirium Tactile and auditory verbal hallucinations Kandisnky–Clerambault's syndrome (delusion of influence, automatism)
*Synthetic psychosis with predominant dissociative reactions* induced by NPS mainly acting on GLU pathways	Dissociation, derealization Somatopsychic depersonalization Illusions, perception distortions Bodily and kinesthetic hallucinations "Near-death" experiences Demonic possession experiences Affective blunting, anhedonia, anesthesia Ekbom syndrome (delusions of infestation, delusional parasitosis) Capgras syndrome (delusional belief that someone known has been replaced by an imposter, delusional misidentification syndrome) Cotard syndrome (delusional belief to be already dead, to not exist, to be putrefied, to have lost own blood or internal organs)
*Synthetic psychosis with predominant paranoia and auditory hallucinations* induced by NPS mainly acting on DA pathways	Paranoia thought Auditory hallucinations Delusions of reference, persecution, grandeur, and jealousy Hypomanic states Aggressiveness and irritability Dysphoria, anxiety, and panic
*Synthetic psychosis with predominant hallucinatory symptoms* induced by NPS mainly acting on SER pathways	Anxiety, irrational fear, and psychic hyperesthesia Acute verbal hallucinosis ± sensual delusion Visual hallucinations with vivid colors associated with intense emotional experiences (either positive or negative) (Eventually kinesthetic and/or tactile hallucinations) Auditory, olfactory, and gustatory hallucinations uncommon (Eventually paranoid delusions, with religious content) (Eventually hypomanic state, suicidal thoughts, and depression)
*Synthetic psychosis with predominant affective-delusional symptoms* induced by NPS mainly acting on SER and DA pathways	Sensation of fear Delusional ideas of interpretation and reference Persecutory, philosophical, and esoteric delusion In some cases, manioform statements
*Synthetic psychosis with predominant mental automatism* induced by NPS mainly acting on mixed pathways	Emotional tension Severe anxiety and confusion Delusional mood, agitation, and delusional persecutory ideas Mental, motor, and senestopathic automatisms Thought broadcasting, thought insertion, or withdrawal Speech automatism Kandinsky–Clerambault's syndrome with verbal hallucinations, delusion of influence and persecutory delusion, as well as automatism

GABA, gamma-aminobutyric acid; Ach, acetylcholine/cholinergic; DA, dopaminergic; SER, serotoninergic; Glu, glutammatergic; NA, noradrenergic.

Overall, after an acute or repeated consumption of SC, neurological toxidromes have been described, such as mental status changes, panic attacks, agitation, aggressiveness, memory distortions, depersonalization, dissociation, catatonia, recurrent psychotic episodes (e.g., delusional thoughts and paranoia), and auditory and/or visual hallucinations (1, 7, 96, 116–121). Psychosis developing in the context of a SC intake and/or intoxication is often described as endoformic (e.g., verbal hallucinations, kinesthetic automatisms, and delusion of grandeur or influence can be present), long-lasting (from 10–14 days to 4–6 weeks), and gradually self-resolve with the persistence of asthenic-depressive symptomatology and cognitive deterioration for more than 4–8 weeks (7, 38, 44, 87). However, several findings documented here show that SC-induced psychoses may persist even in those subjects without a previous history of mental illness and may induce the development of a schizophrenia-like symptomatology, named “Spiceophrenia” (7, 25, 111, 122–125). In addition, there is evidence of a worsening/recrudescence of a mental health disorder (i.e., mainly an affective and/or a psychotic disorder) in those subjects with a preexisting mental condition (29, 34, 35, 50, 56–59, 61, 67, 69, 80, 84, 85, 111, 122). Some studies reported that SC may influence psychiatric course and prognosis, depending on the first age of SC exposure, psychiatric vulnerability/predisposition, a history of a childhood trauma or other traumatic experiences, and specific genetic factors (67, 69, 124). Furthermore, SC have been supposed to determine a more severe psychosis, accompanied with agitation and significant sympathomimetic effects, compared to “classical” cannabis as SC are more potent full receptor agonists at cannabinoid receptors and do not contain cannabidiol, which possesses anxiolytic and antipsychotic properties (1, 7, 70, 126). Despite the evidence presented here, some authors maintain that it is difficult to clearly prove a causal linkage between SC intake and the onset of an *ex novo* psychosis in psychosis-prone/vulnerable subjects and/or the exacerbation of a prodromal psychotic syndrome/appearance of basic symptoms (127). From a therapeutic perspective, benzodiazepines are useful for managing anxiety, agitation, and seizure risk, together with a supportive/symptomatic therapy (1, 7, 30, 32, 36, 39, 41, 46, 48, 59–61, 71, 79, 81). Olanzapine, clozapine, quetiapine, and aripiprazole represent the main prescribed antipsychotic treatments in SC-induced psychoses (1, 7, 37, 50, 57, 60, 62, 65–67, 70, 75, 85, 86), while haloperidol, risperidone, and paliperidone have been prescribed/used in isolated cases (45, 53, 63, 73, 85).

In contrast, published findings reported that synthetic cathinones may cause variable effects in patients with SMI, such as acute psychosis, agitation, violent behavior, confusion, disorientation, insomnia, suicidality, mood lability, and instability in patients affected with bipolar disorder and/or substance poly abuse, while tangential thought process, disorganized speech and behavior, paranoid delusions, and auditory and visual hallucinations are demonstrated in patients affected with schizophrenia, associated with amnesia surrounding their psychotic breaks (88, 90, 91, 93, 94, 96, 100–102, 104, 106, 128). Treatment should be single-minded on managing agitation and psychosis and in supporting renal perfusion. Sedation may be required if the patient is markedly agitated and at risk of self-harm or harm other patients or health professionals (1, 92, 95, 97, 98, 103, 104). In particular, benzodiazepines should be preferred to manage physical violence, to decrease tachycardia and blood hypertension, to prevent seizures, and to reduce muscle hyperactivity, rhabdomyolysis, and renal failure (1). Use of antipsychotics alone should be considered as second-line strategy for managing agitation due to the higher risk in lowering seizure threshold and the higher risk in contributing to the acute toxicity of synthetic cathinones (1, 88, 97, 98, 106). Alternatively, anticonvulsants/mood stabilizers have been suggested (99).

The present review has several limitations which may restrict the generalizability of the findings presented here, e.g., most studies considered here have mainly focused on synthetic cannabinoids which represent the main representative NPS group, but do not necessarily represent all NPS-induced synthetic psychoses. In addition, most studies are represented by case reports, which, on the one hand, may represent a good tool/vehicle to provide more detailed psychopathological and clinical features on NPS consumption and NPS-induced psychosis, but it may not be greatly generalizable to a more representative clinical sample. Most studies do not report if NPS intake is acute or chronic and single or repeated, or do not clearly specify NPS dosage, route of administration, comorbid use of other substances, and so on. Furthermore, the present overview does not possess the scientific robustness of a systematic literature review as most studies are poorly comparable with heterogeneous outcomes and measures. Finally, from a therapeutic point of view, not all studies here considered provide data on therapeutic management and/or strategies. Moreover, those studies providing therapeutic management do not always report dosage(s) and/or treatment duration.

## Conclusion

The constantly and rapidly developing NPS drug scenario represents a challenge for public health, especially so for the field of addiction and mental health. Indeed, NPS consumption is usually associated with an imbalance of a plethora of neurotransmitter pathways and brain receptors, and, hence, correlated to a huge repertory of psychopathological trajectories. Vulnerable individuals (e.g., children, adolescents, and subjects affected with a psychiatric disorder) may be more prone to develop and/or worsen a psychopathological condition, particularly a psychosis which may have peculiar features, from a psychopathological and phenomenological point of view. Due to a great range of medical and psychopathological disturbances associated with NPS intake, it is detrimental for mental health professionals not to be ignorant about the effects, safety, and toxicity profile of NPS so far known, especially the most widespread ones discussed here, e.g., SC and synthetic cathinones. Indeed, further researches should better investigate their clinical and pharmacological knowledge so that better personalized management and treatment strategies/guidelines can be written and disseminated.

## Author Contributions

All authors listed have made a substantial direct and intellectual contribution to the work and approved it for publication. LO and SC collected, analyzed and interpreted data. LO, SC, together with JC and DP drafted the article. DD and FS revised it critically for important intellectual contents.

## Funding

This paper was supported in part by grants of the European Commission (Drug Prevention and Information Programme 2014-16; contract no. JUST/2013/DPIP/AG/4823; *EU-MADNESS project*).

Further financial support was provided by the EU Commission-targeted call on cross border law enforcement cooperation in the field of drug trafficking - DG Justice/DG Migrations and Home Affairs (JUST/2013/ISEC/DRUGS/AG/6429) *Project EPS/NPS* (Enhancing Police Skills concerning Novel Psychoactive Substances; NPS).

## Conflict of Interest

The authors declare that the research was conducted in the absence of any commercial or financial relationships that could be construed as a potential conflict of interest.
